# Celluloid

**Published:** 1876-07

**Authors:** J. H. Mease

**Affiliations:** Reading, Pa.


### ARTICLE V.

### *Celluloid.*

Read before the Central Pennsylvania Dental Association.

BY J. H. MEASE, D. D. S., READING, PA.

The subject allotted to me is, strictly speaking, a part of Mechanical Dentistry, and is generally overlooked or forgotten by dental associations of the present day. Operative dentistry is the sole topic with members who have their profession sufficiently at heart to take part in dental associations

Mechanical dentistry is declining as an art, both in the estimation of the public and the profession. If the public is not able to see the cause of this change, the profession, I feel sure, will agree with me, that the introduction and use of vulcanite, as a base for artificial dentures, with its attending evils, has had a tendency to rapidly bring the art down to a mere trade, and has brought an influx of men, who are often a reproach to the calling; men who have entered the profession without either the cost of time or money, their only ambition appearing to be to manufacture the greatest number of sets of artificial teeth with the least expenditure of time and thought. Their names appear in glaring letters in every newspaper and on every bulletin-board. They float their banners with such inscriptions as, "Excelsior!" "Rubber plates made a specialty," etc.; and if this is not sufficient to attract suffering humanity to their doors, they consider it their duty to sally forth through every town and hamlet, and endeavor to persuade the people to have their natural teeth extracted, or, more appropriately, sacrificed; and in this wise extort money from the unthinking, by making rubber sets to replace the natural organs sacrificed, all the work being guaranteed to last a lifetime. And some of these benefactors, after gleaning one or more harvests from a certain neighborhood, give it a wide birth; in some instances being not unlike the snow in March—they suddenly disappear. The fact that rubber is much cheaper when compared with most bases for artificial teeth, requiring less labor or skill in its manipulation, together with the fact that the public labors under the impression that rubber is pliable in its nature, and would make a more desirable plate to wear in the mouth than any other substance, give it wings to spread over our country like a storm, well nigh obliterating all other bases. And while it thus flourished it created the class of men already alluded to, who sacrificed the natural teeth at such a rate as to cause fear that they would make the American people an edentated race.

The result has been that the field is almost entirely abandoned to those who have no true conception of what belongs to an art calling. Those members of the profession who have the training and the ability to develop the true artistic spirit of mechanical Dentistry have one by one been enlisted into other departments of our calling, and, very naturally, they have ceased to discuss this subject in their meetings and in their journals. They decline to put themselves in competition with men whom they utterly despise; nor are they willing to waste their time and skill upon a substance as faulty and perishable in its nature as rubber has proved itself to be, after a fair and impartial test. A true artist, in order to call forth his best efforts and to attain to any degree of perfection, must constantly have before him the idea that his work will be lasting, and that *no expenditure* of time and care is ever wasted.

The thought that a set of artificial teeth cannot be of any permanent value, necessarily induces a hurried and careless habit of manipulation, and a consequent lowering of the art. During all this time some of the best minds of the profession never ceased to experiment and investigate, for the purpose of removing the objectionable qualities of rubber, or to find a new base that should contain some of the valuable features of rubber, minus the bad ones. After many failures and disappointments to those interested in this laudable enterprise, rays of hope at last burst from an unexpected and mysterious source, when the substance known as cellulose or celluloid was discovered. But the profession, in general, was slow to take hold of this new base, owing, no doubt, to the fact that new things in the profession were not uncommon, and that those who hailed them frequently came to sorrow. Moreover any new invention in this department was sure to meet with powerful opposition from that obstinate and pertinacious monster known as the "Goodyear Dental Vulcanite Company," which not only attempted to defy the dental profession, but to blindfold our highest courts of justice with its pretended claims of original invention. Under such embarrassing circumstances it is not to be wondered at that celluloid, as a base for artificial teeth, was cautiously adopted, even by a few, with the hope and expectation of finding in it something intrinsically better than the rubber plates so universally made and worn. The beauty of the material, its elegant appearance, its simulation fo a gum color, allowing plain teeth to be used in every case, are truly advantages so great as to tempt one to overlook any defects that may arise in its durability or in its working qualities.

The hope may be entertained, with an assurance of its fulfillment, that whatever defect may yet exist, time and experience will put to flight.

Valuable improvements have been made by the inventors in the shapes and qualities of plates, as well as in the apparatus used. The oil apparatus was a disagreeable as well as dangerous process, rendered so by having fire under an open vessel of oil, and the plates frequently came out porous. The first two objections have been removed by substituting glycerine for oil ; but the latter has rather been increased by the change. Some claim to have succeeded with either process, and still continue in its use ; while others use the oil apparatus without a liquid of any kind, and obtain good results.

Steam was next called into requisition, and proved a decided advantage over the two processes first cited, being far more cleanly and certain in its results.

It seems almost impossible to produce a porous plate with steam, and the danger that some fear in the use of this apparatus does not really exist, as the writer, on two several occasions, was called away to the operating chair, leaving the apparatus in full operation in another room, until all the water had blown off in the form of steam, without any bad results.

In working celluloid by steams, pressure should not be made till the steam blows off with some degree of force on raising the safety-valve, as otherwise the plaster model is likely to be injured in bringing the sections of the flask together.

In cases of an undercut model the "practical device," described and illustrated bv cuts in the March number of the *Dental Cosmos* for 1875, by Prof. Wildman, will obviate the difficulty that generally exists in such cases.

The plaster model and investment can be improved in strength by placing the sections of the flask on a warm stove for a few hours. The stove should not be too hot, or else the water in the plaster will be turned into steam and cause an explosion.

In moulding celluloid, very much depends upon how the investment of the teeth and model is made. In the upper investment, or the one embracing the teeth, the plaster should cover or embrace the whole of the try-plate over the alveolar ridge. The vent for the surplus material should be made in the section containing the model. Care should be taken not to extend them so far as to allow the material to flow between the edges of the flask, and prevent it from closing properly.

After opening the flask, and it is found that the plate was not heavy enough to fill out the space, or did not spread properly, place a sheet of tin or lead of the required thickness on the lingual side of the plate, and bring the sections together as before. This plan is also applicable in temporary plates that have been worn till they become loose by absorption.

The difficulty experienced by some in having dark lines or fissures around the air-chamber, can be obviated in most cases by leaving away the air-chamber till the plate is moulded. If there is any occasion to have an air-chamber, it can readily be made by means of the burring engine, or with an enamel chisel by hand. The plate selected should be amply large; if the shape is not well adapted, can readily be changed by immersing it in hot water for a few moments. In some cases it will be necessary to resort to the file and saw.

The advocates of the dry apparatus or heaters claim the following advantages over other methods: That it drives off any excess of camphor remaining in the compound; and that it admits of the patching or repairing of plates better than by the use of oil or glycerine, or by the ordinary steam apparatus.

In summing up: Another line of demarkation between rubber and its rival, celluloid, seems necessary: First, in point of beauty, celluloid stands far superior; and in point of strength we are convinced, from experience, that it has the advantage over rubber, particularly in the event that plate must be heated the second time, which, as is well known, leaves a brittle rubber plate while celluloid is not affected in the least.

In its close adaptation to the teeth, it has a decided advantage over rubber; so, also, in cleanliness and healthfulness, as no sailvation of the person wearing the plate, nor poisoning of the person manufacturing it, can occur from the fumes arising while vulcanizing; and, lastly, no danger from explosion.

Before dismissing the subject, let me urge upon those members who have not already adopted celluloid, to do so at once, and my word for it, after a fair and untiring perseverance, success will crown their efforts. That failure of some kind or other will occur sometimes, is to be expected, as long as man remains a fallible creature —*Penn. Journal of Dental Science.*

